# Morpho-Phylogenetic Evidence Reveals *Neokeissleriella* gen. nov. and Three Novel Species of Lentitheciaceae from Grasses (Poaceae)

**DOI:** 10.3390/jof12010012

**Published:** 2025-12-24

**Authors:** Yong-Xiu Yu, Asha J. Dissanayake, Jian-Kui Liu

**Affiliations:** The Clinical Hospital of Chengdu Brain Science Institute, School of Life Science and Technology, University of Electronic Science and Technology of China, Chengdu 611731, China; yuyongxiu0617@163.com (Y.-X.Y.); asha.dissanayake@uestc.edu.cn (A.J.D.)

**Keywords:** 4 new taxa, Dothideomycetes, *Keissleriella*, multi-locus, taxonomy, phylogeny

## Abstract

The Poaceae family, commonly known as grasses, is one of the most strategically important plant groups on earth. They are globally distributed, found in virtually every terrestrial habitat on earth, from deserts and grasslands to forests and wetlands. An investigation was conducted on the fungi associated with grasses in Sichuan and Guizhou Provinces, China. Based on morphological characteristics and multi-locus phylogenetic analyses (from Maximum Likelihood (ML) and Bayesian Inference (BI)) of combined LSU, SSU, ITS, and *tef1-α* sequence data, a new genus, *Neokeissleriella,* and three novel species—*Keissleriella guttata*, *K. sichuanensis*, and *Neokeissleriella fusispora*—were introduced. Two new host records of *Keissleriella caraganae*, *K. yunnanensis* and a new geographical record of *K. gloeospora* in Sichuan Province, China, are reported. To substantiate the newly established taxa, detailed morphological descriptions and illustrations are provided and supported with molecular phylogenetic analysis. This study broadens the understanding of fungal diversity on Poaceae hosts in Sichuan and Guizhou provinces, China, revealing the high potential for identifying novel taxa.

## 1. Introduction

Poaceae is the most economically valuable crop family among seed plants, comprising nearly 11,800 species among 791 genera [1]. It serves as a primary source of food for humans and feed for livestock, as well as an important raw material for starch processing, sugar production, brewing, papermaking, weaving, and construction. Furthermore, it plays a crucial role in maintaining ecosystem stability [2]. Due to their cosmopolitan distribution and unique ecology, grasses sustain fungi that exhibit a broad spectrum of nutritional modes [3] as commensals, saprobes, and pathogens. Hence, grass fungi drew much attention from researchers. In their work, Thambugala et al. [3] identified 50 taxa on grasses (Poaceae and Gramineae), thereby providing new insights into the taxonomy and diversity of grass-inhabiting fungal species. However, to date, a comprehensive worldwide account for grass fungi is still lacking. On grasses, Dothideomycetes exist as endophytes, saprobes, or pathogens. For example, *Alternaria* Nees, *Bipolaris* Shoemaker, *Parastagonospora* Quaedvlieg, *Pseudoseptoria* Speg, and *Stagonospora* (Sacc.) Sacc. are economically significant pathogens, many of which are notably associated with diseases of Poaceae [4,5,6]. Furthermore, members of Dothideomycetes (e.g., *Bambusicola* D.Q. Dai & K.D. Hyde, *Neokalmusia* Ariyaw. & K.D. Hyde, *Phaeosphaeria* I. Miyake, and *Poaceicola* W.J. Li et al.) are commonly found on Poaceae hosts [7,8,9,10].

Pleosporales, the most significant and largest order in Dothideomycetes, is a taxonomically diverse fungal group, comprising approximately 91 families and 632 genera [11,12]. Lentitheciaceae, which is a monophyletic clade in Pleosporales [13,14], was established in 2009 [15] based on *Lentithecium fluviatile* (Aptroot & Van Ryck) K.D. Hyde, J. Fourn. & Ying Zhang. Members of Lentitheciaceae possess globose ascomata (brown setae or glabrous) and cylindrical to clavate asci with short pedicels. Their ascospores are morphologically diverse, typically fusiform, hyaline to brown, and 1–3-septate (aseptate or muriform in some species). In some cases, they are filiform, fasciculate, and surrounded by the mucilaginous sheath. The asexual morphs exhibit stagonospora-like or dendrophoma-like [16]. Most genera in this group possess a sexual morph, with the exception of *Phragmocamarosporium* [17] and *Towyspora* [18], which are known exclusively from their coelomycetous asexual stages.

Although over 150 species have been reported in Lentitheciaceae, the latest Outline of Fungi [19] indicates that this family currently accommodates only 20 genera (*Crassoascoma* [20], *Darksidea* [21], *Groenewaldia* [22], *Halobyssothecium* [23], *Katumotoa* [24], *Keissleriella* [25], *Lentithecium* [26], *Murilentithecium* [13], *Neolentithecia* [27], *Neoophiosphaerella* [14], *Paralentithecium* [28], *Phragmocamarosporium* [17], *Pleurophoma* [29,30], *Poaceascoma* [31], *Pseudokeissleriella* [32], *Pseudomurilentithecium* [33], *Pseudosetoseptoria* [34], *Setoseptoria* [6], *Tingoldiago* [35], and *Towyspora* [18]). *Keissleriella*, which was established by Höhnel [25] with *K. aesculi* (Höhn.) Höhn as the type species, possesses globose to subglobose ascomata, featuring papillate ostioles that are covered with blackish setae. It produces cylindric-clavate, bitunicate, and fissitunicate asci, which are mostly 8-spored. The ascospores are hyaline to light yellowish brown, ellipsoidal or fusoid, septate, with or without mucilaginous sheaths [36,37,38,39]. Most species in this genus exhibit the sexual morph, while their asexual morph is “Dendrophoma”-like [40]. According to Species Fungorum (http://www.speciesfungorum.org/Names/Names.asp; accessed on 25 October 2025), *Keissleriella* is the most species-rich genus in Lentitheciaceae, comprising 48 epithets. Of these, 40 species have associated morphological data, while only 27 species have molecular data.

The taxonomic status of *Keissleriella* remains controversial. Munk [41] originally distinguished *Keissleriella* from *Trichometasphaeria* based on its host preferences and ascospore characteristics and later placed both genera within the Massarinaceae [42,43]. The synonymy of *Trichometasphaeria* under *Keissleriella*, proposed by Bose [44], was widely adopted in subsequent taxonomic works [14,45,46,47]. However, not all researchers concurred with this opinion. Barr [43] suggested that these two genera should be separated due to the variances in peridium and pseudoparaphyses of their type species. As a result, *Trichometasphaeria* and *Keissleriella* were accommodated in Lophiostomataceae (Pleosporales) and Melanommataceae (Melanommatales), respectively [43]. *Keissleriella* was reclassified within the Massarinaceae by Lumbsch and Huhndorf [48]. With the establishment of Lentitheciaceae by Zhang et al. [15], *Keissleriella* was assigned to Lentitheciaceae based on phylogenetic evidence. The suggestion of Zhang et al. [15] is now generally accepted and has been confirmed by many subsequent studies [36,37,39,49].

Lentitheciaceae species exhibit remarkable diversity in morphology, ecology, and phylogeny. They are globally distributed and exhibit a wide range of lifestyles. Hence, the primary aim of this investigation was to elucidate the taxonomic diversity and phylogenetic relationships of Lentitheciaceae fungi colonizing grass hosts in southwestern China. This was achieved through a polyphasic approach that integrated detailed morphological examinations with a multi-locus molecular phylogenetic analysis, to accurately delineate species and clarify their taxonomic positions within the family.

## 2. Materials and Methods

### 2.1. Isolation of Fungal Strains and Morphological Characterization

A collection of fungi associated with Poaceae hosts was made in Guizhou and Sichuan provinces during 2023. The specimens from this field collection were stored in ziplock bags or envelopes and transferred to the laboratory. Morphological observations and photography were conducted following Senanayake et al. [50], using a Nikon ECLIPSE E200 stereo microscope and a Nikon ECLIPSE Ni-U compound microscope equipped with a DS-Ri2 digital camera. To obtain pure cultures, single-spore isolations were performed following the protocol of Senanayake et al. [50]. Pure cultures were obtained through monospore isolation. Germinated spores were subcultured onto potato dextrose agar (PDA) and maintained at 25 °C in constant darkness. After 7 d of growth, the colony color was documented as of Raynor [51]. Measurements of 10 conidiomata/ascomata and 30 asci/conidia/ascospores per isolate were made with the Tarosoft (R) Image Framework program v. 0.9.7, following the method of Liu et al. [52]. Additional conidial characteristics, including the shape, color, and the presence of guttules, were documented. Adobe Photoshop CS6 (Adobe Systems Inc., San Jose, CA, USA) was used to process and edit the photographic plates for illustrating fungal structures. The resulting herbarium specimens were deposited in the Herbarium of Cryptogams at the Kunming Institute of Botany, Academia Sinica (KUN-HKAS), Kunming, China, and at the Herbarium of the University of Electronic Science and Technology (HUEST), Chengdu, China, respectively. The fungal isolates obtained in this study were deposited in the China General Microbiological Culture Collection Center (CGMCC), Beijing, and in the University of Electronic Science and Technology Culture Collection (UESTCC), Chengdu, respectively. The names of the newly described taxa were formally registered in MycoBank [53].

### 2.2. DNA Extraction, PCR Amplification, and Sequencing

Using the Trelief™ Plant Genomic DNA Kit (Beijing TsingKe Biotech Co., Ltd., China) per the manufacturer’s protocol, total genomic DNA was obtained from 7 d old fungal cultures. The amplification of specific DNA loci was carried out via polymerase chain reaction (PCR). This study utilized four partial gene regions: the large subunit of the nuclear ribosomal RNA gene (LSU), the small subunit of the nuclear ribosomal RNA gene (SSU), the nuclear ribosomal internal transcribed spacer (ITS: ITS1-5.8S-ITS2), and the translation elongation factor 1-alpha (*tef1-α*). The LSU, SSU, ITS, and *tef1-α* gene regions were amplified with the primer pairs LR0R/LR5, NS1/NS4, ITS5/ITS4 and 983F/2218R, respectively [54,55,56,57]. The PCR mixture (50 µL final volume) consisted of 25 µL 2×Taq Plus MasterMix (Dye) (CoWin Biosciences, (Taizhou), Co., Ltd., Taizhou, China), 19 µL ddH_2_O, 2 µL DNA template, and 2 µL of each primer (10 µM/L). The PCR thermal cycle program for LSU, SSU, ITS, and *tef1-α* amplification was as follows: an initial denaturing step of 94 °C for 5 min, followed by 35 cycles of denaturation at 94 °C for 30 s, annealing at 56 °C (LSU, SSU, ITS, *tef1-α*) for 30 s, elongation at 72 °C for 30 s, and a final extension at 72 °C for 10 min. The amplification results were confirmed via 1% agarose gel electrophoresis using GelRed for visualization. DNA sequencing was performed by Beijing Tsingke Biotechnology Co., Ltd. (Chengdu, China) using the primers listed above.

### 2.3. Phylogenetic Analysis

A BLAST (Basic Local Alignment Search Tool, https://blast.ncbi.nlm.nih.gov/Blast.cgi, accessed on 10 October 2025) search was conducted for each obtained sequence within the NCBI database to assess sequence similarity and aid in preliminary identification. The phylogenetic analysis was based on a concatenated alignment of the LSU, SSU, ITS, and *tef1-α*, which included sequences from reference taxa downloaded from GenBank (Table 1). The sequences were aligned by the online multiple alignment program MAFFT v.7 (http://mafft.cbrc.jp/alignment/server/, accessed on 10 October 2025) [58], and the alignment was manually optimized in BioEdit v.7.0.9 [59]. The four-gene dataset was concatenated by Mesquite v. 3.11 (http://www.mesquiteproject.org/, accessed on 10 October 2025) for multi-gene phylogenetic analysis. Phylogenetic analyses were conducted using both Maximum Likelihood (ML) and Bayesian Inference (BI) methods, as described by Dissanayake et al. [60]. The Maximum Likelihood (ML) analysis was conducted with RAxML-HPC v.8 on the CIPRES Science Gateway V3.3 (https://www.phylo.org/portal2/home.action, accessed on 10 October 2025), employing rapid bootstrapping. The substitution model was set to GTR with a discrete GAMMA distribution. The Bayesian inference (BI) analysis was performed using MrBayes v. 3.1.2 [61,62]. The optimal substitution model for each gene partition was selected using MrModeltest 2.3 [63]. Subsequently, the posterior probabilities were computed in MrBayes v.3.1.2 [61,62] through Markov Chain Monte Carlo (MCMC) sampling. Two software packages were employed for the presentation of phylogenetic trees. FigTree v.1.4.4 (http://tree.bio.ed.ac.uk/software/figtree/, accessed on 10 October 2025) was used for the initial visualization and analysis, while Adobe Illustrator 22.1 was applied for the final esthetic refinement and layout. The phylogenetic data (alignment and tree files) are publicly available in TreeBASE under submission ID: 32437 (TreeBASE 2025).

## 3. Results

### 3.1. Phylogenetic Analysis

**Figure 1:**
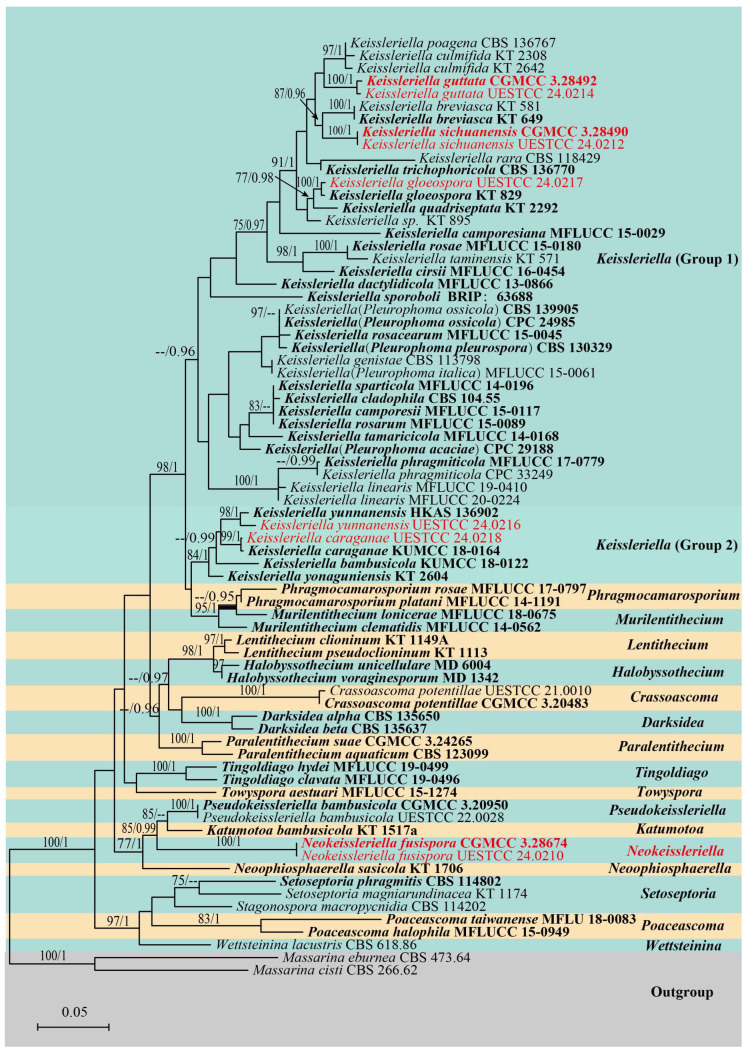
Phylogeny of Lentitheciaceae from combined LSU, SSU, ITS, and *tef*1-α data. Both ML bootstrap supports ≥75% and Bayesian posterior probabilities (PP) ≥ 0.95 from MCMC analysis are denoted above the branches. Newly obtained isolates are denoted in red, and ex-type strains are marked in bold.

The dataset for phylogenetic analysis comprised a concatenated sequence alignment of the LSU, SSU, ITS, and *tef*1-α loci from 74 taxa. The tree was rooted using *Massarina cisti* (CBS 266.62) and *M. eburnea* (CBS 473.64) as outgroups. The concatenated matrix comprised a total of 3315 characters (LSU: 1–851 bp; SSU: 852–1867 bp; ITS: 1868–2389 bp; *tef1-α*: 2390–3315 bp), including gaps. The phylogenetic trees generated from Maximum Likelihood (ML) and Bayesian Inference (BI) analyses showed generally congruent topologies. The best-scoring maximum likelihood tree (Figure 1) represents the phylogenetic relationships with a final likelihood value of—18,113.659579. The GTR+I+G model was selected as the best-fit evolutionary model for all loci (LSU, SSU, ITS, and *tef1-α*) in the Bayesian analysis. Six Markov chains were run simultaneously for 1,645,000 generations, with trees sampled at every 1000th generation, yielding a total of 1645 trees. After discarding the first 329 trees as burn-in, the remaining 1316 trees were used to calculate the posterior probabilities for the majority-rule consensus tree, with a convergence diagnostic critical value of 0.01.

The analysis result (Figure 1) comprises 62 representative species of Lentitheciaceae. Two isolates (CGMCC 3.28674, UESTCC 24.0210) obtained from grasses formed a distinct clade in Lentitheciaceae closer to the clade of *Katumotoa bambusicola* (KT 1517a), *Pseudokeissleriella bambusicola* (CGMCC 3.20950), and *Neoophiosphaerella sasicola* (KT 1706), with poor statistical support, and were identified as a new genus: *Neokeissleriella* viz. *N. fusispora*. Another two isolates were identified as *K. guttata* (CGMCC 3.28492, UESTCC 24.0214), which formed an independent sister clade to *K. culmifida* (KT 2642) and *K. poagena* (CBS 136767), with the support of (100% ML/1.00 PP). The new isolates of *K. sichuanensis* (CGMCC 3.28490, UESTCC 24.0212) formed a distinct clade sister to *K. breviasca* (KT 649, KT 581), while another strain of *K. yunnanensis* (UESTCC 24.0216) formed a strong (98% ML/1.00 PP) clade to its type strain *K. yunnanensis* (HKAS 136902). The phylogeny showed that the two species, *Keissleriella gloeospora* (UESTCC 24.0217) and *K. caraganae* (UESTCC 24.0218), formed two distinct strong clades (100% ML/1.00 PP and 99% ML/1.00 PP) with the type isolates of *K*. *gloeospora* (KT 829) and *K*. *caraganae* (KUMCC 18-0164), respectively.

### 3.2. Taxonomy

***Neokeissleriella*** Y. Xiu Yu & Jian K. Liu, gen. nov.

MycoBank: MB 857721

Etymology: The prefix “neo-“means “new”. “*Neokeissleriella*” refers to its morphological similarity to the genus “*Keissleriella*”.

Saprobic on dead stems. Sexual morph: *Ascomata* immersed to erumpent, subglobose, dark brown to black, unilocular, coriaceous, glabrous, with a distinct, dark brown ostiole. *Peridium* consists of multiple layers of hyaline to brown cells, forming a *textura angularis. Hamathecium* pseudoparaphyses, remotely septate. *Asci* 8-spored, cylindrical to cylindric-clavate, bitunicate, short pedicellate, with an ocular chamber. *Ascospores* overlapping bi-seriate, fusiform, tapering to subobtuse ends; the upper cell is swollen towards the median septum, hyaline, septate, guttulate, and without a mucilaginous sheath. Asexual morph: Undetermined.

Type species—***Neokeissleriella fusispora*** Y. Xiu Yu & Jian K. Liu.

Notes*:* The multi-locus phylogenetic analysis revealed that our new isolates of *Neokeissleriella fusispora* (CGMCC 3.28674, UESTCC 24.0210) form a distinct, monophyletic clade within Lentitheciaceae. As shown in Figure 1, this clade clustered with the monotypic genera *Katumotoa* (type: *Ka. bambusicola*), *Pseudokeissleriella* (type: *P. bambusicola*), and *Neoophiosphaerella* (type: *N. sasicola*). Though *Neokeissleriella* shares immersed, subglobose ascomata with *Katumotoa*, it differs significantly in ascospore morphology. *Neokeissleriella fusispora* has fusiform ascospores tapering to subobtuse ends, with a swollen upper cell near the median septum. In contrast, *Katumotoa* is characterized by apiosporous ascospores that feature elongated bipolar mucilaginous sheaths [24]. *Neokeissleriella fusispora* is morphologically similar to *Pseudokeissleriella*, particularly in sharing immersed, globose ascomata and fusiform ascospores. However, *Pseudokeissleriella* (type species *P. bambusicola*) differs in having 1(–3)-septate ascospores surrounded by a mucilaginous sheath [32]. A key distinction between the genera is that *Neokeissleriella* has immersed, globose ascomata and fusiform, 1-septate ascospores, whereas *Neoophiosphaerella* has hemispherical ascomata with clypei and filiform, multi-septate ascospores [14].

*Neokeissleriella fusispora* represents a distinct species from *K. bambusicola*, *P. bambusicola*, and *N. sasicola*, according to the recommendations of Jeewon & Hyde [64], which is supported by the sequence comparisons of LSU (18/818; 15/821; 18/817) and ITS (64/533; 59/489; 59/439) regions. A new genus, *Neokeissleriella*, is proposed within the Lentitheciaceae family based on corroborating evidence from phylogenetic analyses and distinctive morphological characteristics.

**Figure 2:**
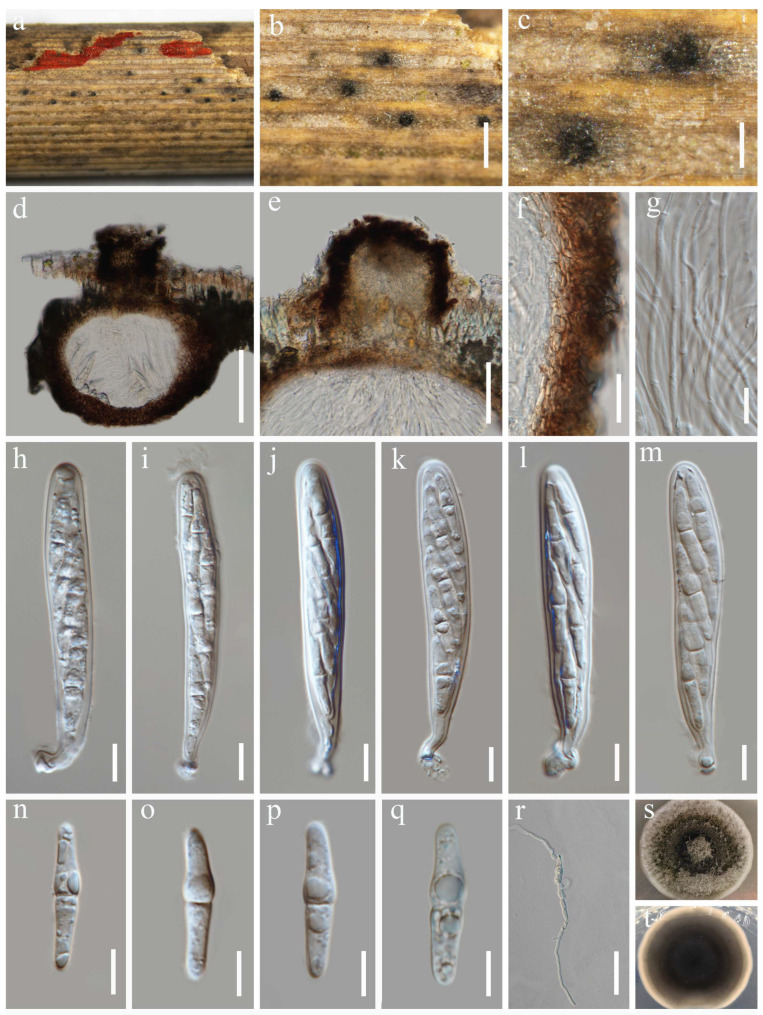
*Neokeissleriella fusispora* (HKAS 144515, holotype). (**a**–**c**) Ascomata on host substrate. (**d**) Vertical section through ascoma. (**e**) Ostiole. (**f**) Structure of the peridium. (**g**) Hamathecium. (**h**–**m**) Asci. (**n**–**q**) Ascospores. (**r**) Germinating ascospore. (**s**,**t**) Colonies on PDA, above (**s**) and below (**t**). Scale bars: (**b**) = 500 µm, (**c**) = 150 µm, (**d**) = 100 µm, (**e**) = 50 µm, (**f**) = 20 µm, (**g**–**q**) = 10 µm, (**r**) = 50 µm.

***Neokeissleriella fusispora*** Y. Xiu Yu & Jian K. Liu, sp. nov. (Figure 2)

MycoBank: MB 857722

Etymology: The epithet “fusispora” refers to the fusiform ascospores.

Holotype: HKAS 144515

Saprobic on dead stems of *Tripidium arundinaceum*. Sexual morph: *Ascomata* 264–384 μm high, 207–410 µm diam. ($\bar{x}$ = 300 × 315 µm, n = 10), visible as dark brown to black, scattered, immersed to slightly erumpent, glabrous, in vertical section subglobose, unilocular, coriaceous, with a central ostiole. *Ostiole* 79–120 µm high, 80–102 μm diam. ($\bar{x}$ = 102 × 89 µm, n =10), dark brown, periphysate. *Peridium* 4–10 µm diam., relatively thin, multi-layered, comprising hyaline to brown cells of *textura angularis*. *Hamathecium* 1.3–3.0 µm wide, comprising numerous, branched, septate, filiform pseudoparaphyses. *Asci* 77–100 × 10–14 µm ($\bar{x}$ = 84.4 × 12 µm, n = 30), 8-spored, bitunicate, fissitunicate, cylindrical to cylindric-clavate, with a short pedicel, apically rounded, with an ocular chamber. *Ascospores* 23–32 × 5–7 µm ($\bar{x}$ = 27 × 5.3 µm, n = 30), overlapping bi-seriate, fusiform, tapering to subobtuse ends, hyaline, 1-septate, the upper cell swollen towards the median septum, straight or slightly curved, smooth-walled, depressed in the middle, and without a mucilaginous sheath. Asexual morph: Undetermined.

Culture characteristics: Germination occurred on PDA within 24 h at 25 °C. Fungal colonies growing on PDA reaching a diam. of 3.2 cm after 7 d at 25 °C in the dark. On PDA, fungal colonies are circular, flat, with an even margin, a velvety surface, and a color gradient from a dark brown center to a white periphery. On the reverse, colonies displayed a dark pigmentation, bordered by a white edge.

Material examined: China, Guizhou Province, Guiyang City, Guiyang Ahahu National Wetland Park, 26°33′50″ N, 106°40′2″ E, 1100 m elevation, on dead stems of *Tripidium arundinaceum*, 04 November 2023, Y. Xiu Yu, (HKAS 144515, **holotype**); ex-type culture CGMCC 3.28674; *ibid*., HUEST 24.0227, isotype, ex-isotype culture UESTCC 24.0210.

**Figure 3:**
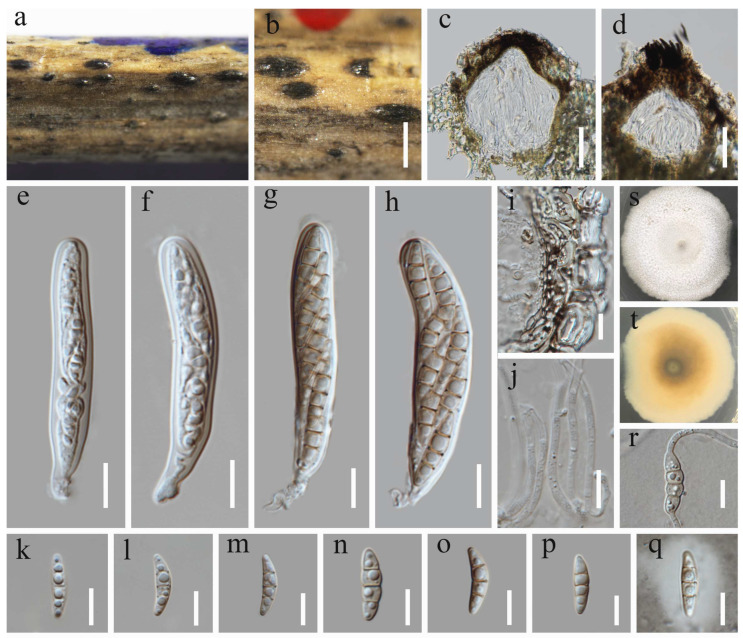
*Keissleriella caraganae* (HUEST 24.0235, new host record). (**a**,**b**) Ascomata on host substrate. (**c**) Vertical section through ascoma. (**d**) Apical setae of ascoma. (**e**–**h**) Asci. (**i**) Structure of the peridium. (**j**) Hamathecium. (**k**–**p**) Ascospores. (**q**) Ascospore immersed in Indian ink. (**r**) Germinating ascospore. (**s**,**t**) Colonies on PDA, above (**s**) and below (**t**). Scale bars: (**b**) = 200 µm, (**c**,**d**) = 50 µm, (**e**–**r**) = 10 µm.

***Keissleriella caraganae*** Chaiwan, Phookamsak, Wanas. & K.D. Hyde, Fungal Diversity (2019) 95:1–273 (Figure 3)

MycoBank: MB 555523

Saprobic on dead stems of grass litter. Sexual morph: *Ascomata* 90–132 µm high, 111–168 µm diam., black dots on host surface, solitary or in groups, scattered, visible as raised, semi-immersed, globose to subglobose, glabrous, ostiolate at center, with minute papilla. *Ostiole* 19–49 µm high, 31–44 μm diam. ($\bar{x}$ = 34 × 37 µm, n = 10), covered with brown, short setae. *Peridium* 5.5–9.5 µm wide, composed of several layers of small, flattened, brown to dark brown pseudoparenchymatous cells, arranged in a *textura angularis* to *textura prismatica*, intermixed with the host cells. *Hamathecium* 1.8–2.3 µm wide, comprising numerous, filiform, branched, septate pseudoparaphyses. *Asci* 52–67 × 8.8–13.3 µm ($\bar{x}$ = 60 × 11 µm, n = 30), 8-spored, bitunicate, fissitunicate, cylindrical to cylindric-clavate, short pedicellate, apically rounded, with a well-developed ocular chamber. *Ascospores* 17–20 × 4.2–5.4 µm ($\bar{x}$ = 19 × 4.7 µm, n = 30), overlapping 1–2-seriate, fusiform to ellipsoid, hyaline or pale yellowish, with rounded ends, (1–)3-septate, slightly constricted at the central septum, smooth-walled, with small guttules, surrounded by mucilaginous sheaths. Asexual morph: Undetermined.

Culture characteristics: Ascospores germinated on PDA within 24 h at 25 °C. After four weeks on PDA at 25 °C in the dark, colonies grow 2.2–2.5 cm in diam. They appear circular, flattened, and dense with concentric zones; the surface is velvety and milky white, being white and less dense at the edges; on the reverse, colonies displayed a pale yellow to milky white coloration.

Material examined: China, Sichuan Province, Mianyang City, Qiqu Mountain. 31°40′23″ N, 105°11′1″ E, 560 m elevation, on dead stems of grass litter, 8 December 2023, Y. Xiu Yu, (HUEST 24.0235), living culture UESTCC 24.0218.

Notes: As revealed by the multi-gene phylogeny (Figure 1), our isolate (UESTCC 24.0218) clustered with the type species *Keissleriella caraganae* (KUMCC 18-0164) with a maximum statistical support (99% ML/1 (PP); see Figure 1). Morphologically, our isolate UESTCC 24.0218 closely resembles the type strain *K. caraganae* (KUMCC 18-0164). Both shared key diagnostic characters [39]: ascomata with an ostiolar neck containing short, brown, aseptate periphyses; bitunicate, broadly cylindrical to cylindric-clavate asci; and fusiform, pale yellowish, septate ascospores with mucilaginous sheaths.

**Figure 4:**
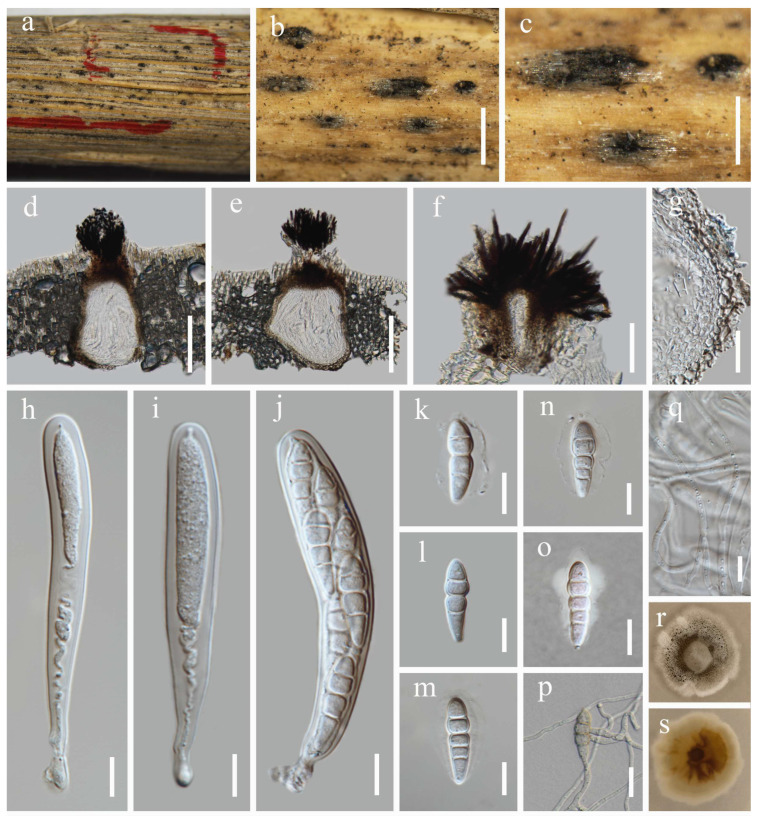
*Keissleriella gloeospora* (HUEST 24.0234, a new geographical record) (**a**–**c**) Ascomata on host substrate. (**d**,**e**) Vertical section through ascoma. (**f**) Apical setae of ascoma. (**g**) Structure of the peridium. (**h**–**j**) Asci. (**k**–**n**) Ascospores. (**o**) Ascospore immersed in Indian ink. (**p**) Germinating ascospore. (**q**) Hamathecium. (**r**,**s**) Colonies on PDA, above (**r**) and below (**s**). Scale bars: (**b**) = 300 µm, (**c**) = 200 µm, (**d**,**e**) = 100 µm, (**f**) = 50 µm, (**g**) = 20 µm, (**h**–**q**) = 10 µm.

***Keissleriella gloeospora*** (Berk. & Curr.) S.K. Bose, Phytopath Z. 41: 190. 1961 (Figure 4)

MycoBank: MB 316017

Saprobic on dead stems of *Tripidium arundinaceum*. Sexual morph**:** *Ascomata* 43–328 µm high, 40–185 µm diam. ($\bar{x}$ = 174 × 109 µm, n = 20), visible as black dots, scattered, solitary or in groups, semi-immersed to immersed, spheroidal to subglobose, papillate, coriaceous, with a central ostiole. *Ostiole* 17–89 µm high, 19–76 μm diam. ($\bar{x}$ = 47 × 46 µm, n = 10), covered with red–brown to dark brown setae. *Peridium* 7–12 µm diam., relatively thin, multi-layered, composed of 3–4 layers of hyaline to brown cells of *textura angularis*. *Hamathecium* 1.9–3.0 µm wide, comprising numerous, branched, septate, filiform pseudoparaphyses. *Asci* 71–95 × 10–14 µm ($\bar{x}$ = 83 × 13 µm, n = 30), 8-spored, bitunicate, fissitunicate, cylindrical to cylindric-clavate, apex round, short stipitate, with a chamber. *Ascospores* 20–32 × 5.7–7.6 µm ($\bar{x}$ = 25 × 6.6 µm, n = 30), arranged biseriately, fusiform to clavate, (-3)4-septate, slightly curved, slightly constricted at the septa, hyaline, surrounded by an entire mucilaginous sheath. Asexual morph: Undetermined.

Culture characteristics: The ascospores germinated on PDA within 24 h at 25 °C. After one week of growth in the dark at 25 °C, the colonies on PDA reached 1.2–1.4 cm in diam. They appeared circular, flattened, and dense, with concentric zonation. The color ranged from pale brown to milky white, featuring velvety, white mycelium at the edges and a denser center. The reverse side was pale brown to milky white.

Material examined: China, Sichuan Province, Chengdu City, Qingshuihe Campus, University of Electronic Science and Technology. 30°45′23″ N, 103°55′25″ E, 520 m elevation, on dead stems of *Tripidium arundinaceum*, 5 October 2023, Y. Xiu Yu, (HUEST 24.0234); living culture UESTCC 24.0217.

Notes: With strong statistical support (100% ML/1(PP); see Figure 1), our isolate (UESTCC 24.0217) grouped with the *Keissleriella gloeospora* type strain (KT 829) in the phylogenetic tree (Figure 1). The BLASTn results for the ITS and LSU sequences of our isolate (UESTCC 24.0217) exhibited a high similarity with the type species *K*. *gloeospora* (KT 829) at 99.88% and 100%. The new collection was identified as *K. gloeospora*, as their morphological characteristics are closely aligned with the descriptions provided by Bose [44] and Shearer et al. [65].

**Figure 5:**
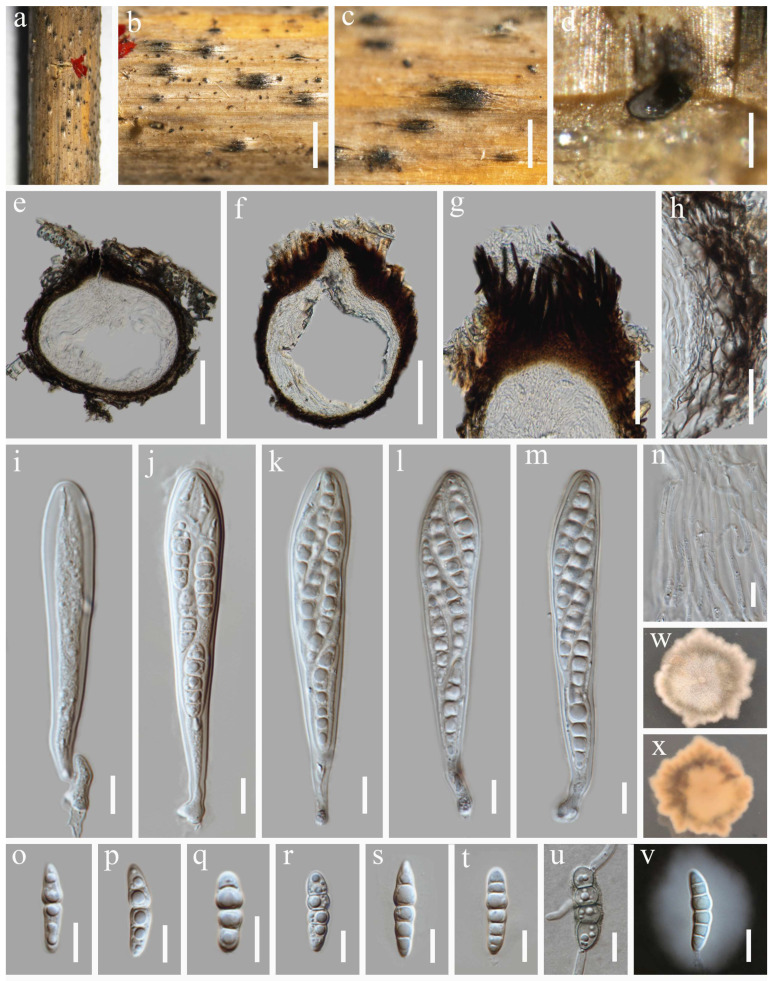
*Keissleriella guttata* (HKAS 144517, holotype). (**a**–**d**) Ascomata on host substrate. (**e**,**f**) Vertical section through ascoma. (**g**) Apical setae of ascoma. (**h**) Structure of the peridium. (**i**–**m**) Asci. (**n**) Hamathecium. (**o**–**t**) Ascospores. (**u**) Germinating ascospore. (**v**) Ascospore immersed in Indian ink. (**w**,**x**) Colonies on PDA, above (**w**) and below (**x**). Scale bars: (**b**) = 500 µm, (**c**) = 250 µm, (**d**) = 200 µm, (**e**,**f**) = 100 µm, (**g**) = 50 µm, (**h**) = 20 µm, (**i**–**v**) = 10 µm.

***Keissleriella guttata*** Y. Xiu Yu & Jian K. Liu, sp. nov. (Figure 5)

MycoBank: MB 857720

Etymology: The epithet “guttata” refers to the characteristic of its hymenium surface.

Holotype: HKAS 144517

Saprobic on dead stems of *Tripidium arundinaceum*. Sexual morph: *Ascomata* 94–321 μm high, 151–261 µm diam. ($\bar{x}$ = 250 × 206 µm, n = 20), visible as black, scattered, globose to pyriform, papillate, unilocular, coriaceous, with a central ostiole. *Ostiole* 36–109 µm high, 26–74 μm diam. ($\bar{x}$ = 61 × 45 µm, n = 10), covered with upright to convergent red-brown to almost black setae. *Peridium* 6.6–12 µm diam., relatively thin, multi-layered, composed of 3–4 layers of hyaline to brown cells of *textura angularis*. *Hamathecium* 1.4–2.8 µm wide, comprising numerous, branched, septate, filiform pseudoparaphyses. *Asci* 75–125 × 12–17 µm ($\bar{x}$ = 93 × 15 µm, n = 30), fissitunicate, cylindrical to cylindric-clavate, apex obtuse, stipitate, 8-spored, bitunicate, with apical chambers. *Ascospores* 17–29 × 4.5–6.8 µm ($\bar{x}$ = 23 × 5.5 µm, n = 30), arranged in biseriate, fusiform, some narrowly ellipsoidal with rounded ends, with a nearly median primary septum, 3- to 5-septate, slightly constricted at the septa, the cell above the primary septum swollen, guttulate, hyaline, smooth, with mucilaginous sheaths. Asexual morph: Undetermined.

Culture characteristics: The ascospores germinated on PDA within 24 h at 25 °C. After four weeks under the same conditions in darkness, the colonies reached only 1.3–1.6 cm in diam. They were irregular in shape with an undulate edge, appearing flattened, dense, and milky white in color. The center was denser than the edge, and on the reverse, it showed a coloration ranging from milky white to pale orange.

Material examined: China, Sichuan Province, Mianyang City, Phoenix Mountain Forest Park. 31°23′51″ N, 104°43′30″ E, 520 m elevation, on dead stems of *Tripidium arundinaceum*, 08 December 2023, Y. Xiu Yu, (HKAS 144517, **holotype**); ex-type culture CGMCC 3.28492; *ibid*., HUEST 24.0231, isotype, ex-isotype culture UESTCC 24.0214.

Notes: The BLASTn results for the ITS sequence of *Keissleriella guttata* (CGMCC 3.28492) exhibited a high similarity with two species: *K. culmifida* (KT 2308) at 97.96% (480/490 bp, 1 gap) and *K. poagena* (CBS 136767) at 97.95% (477/487 bp, 1 gap). The LSU and *tef1-α* BLASTn results showed 99.88% (847/848 bp, 0 gaps) and 96.32% (890/924 bp, 1 gap) matches with *K. culmifida* (KT 2308), respectively. As shown in the multi-gene phylogeny (Figure 1), *K. guttata* (CGMCC 3.28492 and UESTCC 24.0214) forms a sister group with *K. culmifida* and *K. poagena*, indicating a close phylogenetic relationship. Morphologically, *K. guttata* is comparable to *K. culmifida* and *K. poagena* as the latter two possess fusiform ascospores. Ascospores of *K. culmifida* and *K. poagena* are mostly 3-septate (*K. poagena* is morphologically close to *K. culmifida*), while in *K. guttata*, they are often 3-5-septate [14,66]. Accordingly, they are different in terms of the L/W ratio of ascospores (*K. culmifida* L/W 3.9 vs. *K. guttata* L/W 4.1) [14]. Therefore, *Keissleriella guttata* is formally described here as a new species.

**Figure 6:**
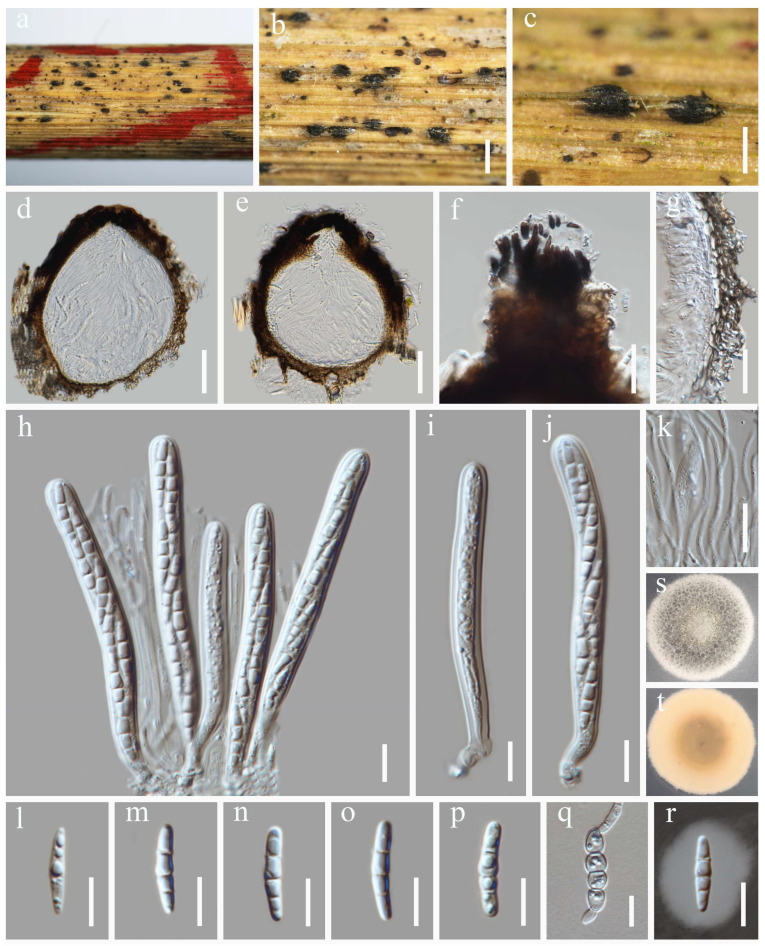
*Keissleriella sichuanensis* (HKAS 144516, holotype). (**a**–**c**) Ascomata on host substrate. (**d**,**e**) Vertical section through ascoma. (**f**) Apical setae of ascoma. (**g**) Structure of the peridium. (**h**–**j**) Asci. (**k**) Hamathecium. (**l**–**p**) Ascospores. (**q**) Germinating ascospore. (**r**) Ascospore immersed in Indian ink. (**s**,**t**) Colonies on PDA, above (**s**) and below (**t**). Scale bars: (**b**) = 300 µm, (**c**) = 200 µm, (**d**,**e**) = 50 µm, (**f**,**g**,**k**) = 20 µm, (**h**–**j**,**l**–**r**) = 10 µm.

***Keissleriella sichuanensis*** Y. Xiu Yu & Jian K. Liu, sp. nov. (Figure 6)

MycoBank: MB 857413

Etymology: The specific epithet refers to Sichuan Province in China, where the holotype was collected.

Holotype: HKAS 144516

Saprobic on dead stems of grass litter. Sexual morph: *Ascomata* 141–232 μm high, 138–205 µm diam. ($\bar{x}$ = 200 × 168 µm, n = 20), scattered, visible as dark brown to black, immersed to slightly erumpent, subglobose, unilocular, coriaceous, with a central ostiole. *Ostiole* 40–67 µm high, 44–54 μm diam. ($\bar{x}$ = 52 × 48 µm, n = 10), covered with dark brown to almost black setae. *Peridium* 3.5–7.4 µm diam., relatively thin, multi-layered, composed of 3–4 layers of hyaline to brown cells of *textura angularis*. *Hamathecium* 1.7–3.4 µm wide, comprising numerous, filiform, branched, septate pseudoparaphyses. *Asci* 70–100 × 7–10 µm ($\bar{x}$ = 82.4 × 8.2 µm, n = 30), 8-spored, bitunicate, fissitunicate, cylindrical to cylindric-clavate, with short pedicels, apically rounded with ocular chambers. *Ascospores* 16–21 × 3–4.3 µm ($\bar{x}$ = 18 × 3.3 µm, n = 30), narrowly fusiform, with a nearly median primary septum, 3-septate (1+1+1), slightly constricted at the septa, hyaline, smooth, with mucilaginous sheathes. Asexual morph: Undetermined.

Culture characteristics: The ascospores germinated on PDA within 24 h at 25 °C. After four weeks of growth under the same conditions in darkness, the colonies reached 2.2–2.5 cm in diam. They were circular, flattened, dense, and concentrically zonate, with colors ranging from milky white to black–gray. The mycelium was velvety and white at the edges, while the center was denser; on the reverse side, it displayed a pale yellow to milky white color.

Material examined: China, Sichuan Province, Mianyang City, Anzhou District, Jinhua village. 31°31′59″ N, 104°14′48″ E, 650 m elevation, on dead stems of grass litter, 07 December 2023, Y. Xiu Yu, (HKAS 144516, **holotype**); ex-type living culture CGMCC 3.28490; *ibid*., HUEST 24.0229, isotype, ex-isotype living culture UESTCC 24.0212.

Notes: The analysis shows that *Keissleriella sichuanensis* (CGMCC 3.28490, UESTC 24-0212) is nested within *Keissleriella* and forms a strong (100% ML) sister clade with *K. breviasca* (KT 649, KT 581) (Figure 1). The ITS sequence of *K. sichuanensis* (CGMCC 3.28490) shared the highest similarity (95.60%; 456/477 bp, 0 gaps) with *K. breviasca* (KT 649) in the BLASTn search. Between the LSU sequences of *K. sichuanensis* (CGMCC 3.28490) and *K. breviasca* (KT 649), a 99.65% identity (848/851 bp, 0 gaps) was revealed. *K. sichuanensis* was morphologically similar to K. breviasca, both characterized by scattered, immersed to slightly erumpent ascomata and smooth, 3-septate, hyaline ascospores with mucilaginous sheaths. However, *K. sichuanensis* can be easily distinguished from *K. breviasca* by 8-spored asci (Figure 6j) (vs. 4-spored asci) and smaller ascospores (av. 18 × 3.3 µm vs. av. 21.1 × 4.3 µm) [14]. Accordingly, we describe *K. sichuanensis* as a new species in the *Keissleriella*.

**Figure 7:**
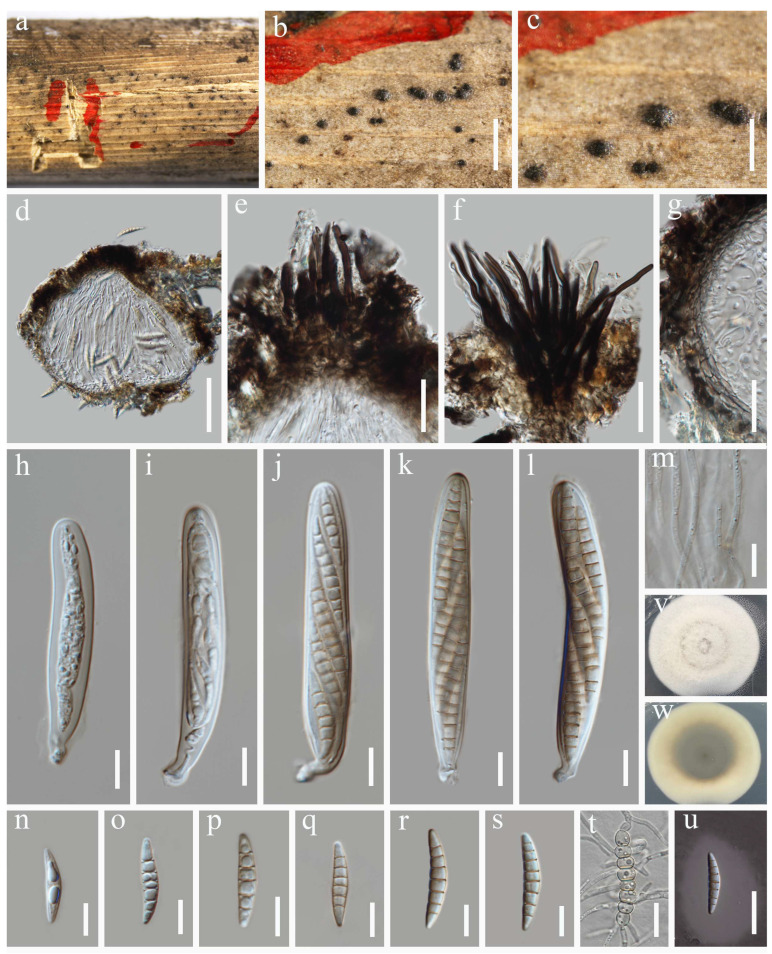
*Keissleriella yunnanensis* (HUEST 24.0233, new host record) (**a**–**c**) Ascomata on host substrate. (**d**) Vertical section through ascoma. (**e**,**f**) Apical setae of ascoma. (**g**) Structure of the peridium. (**h**–**l**) Asci. (**m**) Hamathecium. (**n**–**s**) Ascospores. (**t**) Germinating ascospore. (**u**) Ascospore immersed in Indian ink. (**v**,**w**) Colonies on PDA, above (**v**) and below (**w**). Scale bars: (**c**) = 300 µm, (**b**) = 200 µm, (**d**) = 50 µm, (**e**–**g**,**t**,**u**) = 20 µm, (**h**–**s**) = 10 µm.

***Keissleriella yunnanensis*** Y.R. Sun, Yong Wang bis & K.D. Hyde, Fungal Diversity 2025: 1–201 (Figure 7)

MycoBank: MB 857412

Saprobic on dead stems of *Tripidium arundinaceum*. Sexual morph: *Ascomata* 61–212 µm high, 56–202 µm diam. ($\bar{x}$ = 131 × 119 µm, n = 20), visible as black dots, scattered, solitary or in groups, semi-immersed to immersed, globose to subglobose, papillate, coriaceous, with a central ostiole. *Ostiole* 14–68 µm high, 14–57 μm diam. ($\bar{x}$ = 44 × 38 µm, n = 10), covered with red–brown to dark brown setae. *Peridium* 5.5–8.5 µm diam., relatively thin, multi-layered, composed of 3–4 layers of hyaline to brown cells of *textura angularis*. *Hamathecium* 1.9–3.8 µm wide, comprising numerous, filiform, branched, septate pseudoparaphyses. *Asci* 73–102 × 9–15 µm ($\bar{x}$ = 88 × 12 µm, n = 30), 8-spored, bitunicate, fissitunicate, cylindrical to cylindric-clavate, apex rounded, short stipitate, with apical chambers. *Ascospores* 23–32 × 4.5–6.5 µm ($\bar{x}$ = 27 × 5.4 µm, n = 30), hyaline when young, pale brown when mature, arranged in biseriate, fusiform, with rounded ends, (-5)8-septate, slightly curved, slightly constricted at the septa, smooth, surrounded by mucilaginous sheaths. Asexual morph: Undetermined.

Culture characteristics: The ascospores germinated on PDA within 24 h at 25 °C. After one week under the same conditions in darkness, the colonies reached 1.5–1.6 cm in diam. They are circular, flattened, dense, and concentrically zonate, with a milky white color and velvety mycelium. The center was denser than the edge, and the reverse side appeared gray–green to milky white.

Material examined: China, Sichuan Province, Dujiangyan City, Earthquake relic site of Hongkou. 31°5′14″ N, 103°36′38″ E, 1020 m elevation, on dead stems of *Tripidium arundinaceum*, 16 October 2023, Y. Xiu Yu, (HUEST 24.0233); living culture UESTCC 24.0216.

Notes: As revealed by the multi-gene tree (Figure 1), our new collection (UESTCC 24.0216) grouped with the type of *Keissleriella yunnanensis* (HKAS 136902) in a well-supported clade (98% ML/1.00 PP). The new collection shares morphological similarities with *K. yunnanensis* (HKAS 136902). However, it differs from *K*. *yunnanensis* (HKAS 136902) in having larger asci (73–102 × 9–15 μm vs. 60–80 × 6–8 μm) and ascospores (23–32 × 4.5–6.5 μm vs. 16–20 × 2.5–4.5 μm) [67]. BLASTn results of UESTCC 24.0216 showed 100% identity (491/491 bp, 0 gap) in the ITS region with *K. yunnanensis* (HKAS 136902), and 97.79% similarity (883/903 bp, 0 gap) in the *tef*1-α gene. Therefore, the fungal isolate obtained from *Tripidium arundinaceum* is identified as *K. yunnanensis*.

## 4. Discussion

In recent years, there have been comprehensive taxonomic and phylogenetic investigations on the family Lentitheciaceae by various researchers. However, based on the first multigene phylogeny of Pleosporales, Zhang et al. [15] proposed the family Lentitheciaceae, which initially comprised *Lentithecium*, *Katumotoa*, and *Keissleriella*. Wijayawardene et al. [68] subsequently adopted the taxonomic framework for the family, which comprised ten genera as established by Dong et al. [69]. Previous morphological and phylogenetic studies have revealed that the identification of *Lentithecium*, *Keissleriella*, and *Setoseptoria* is confusing, with several taxa having problematic placements and being transferred across different genera. For example, although Suetrong et al. [70] phylogenetically transferred *Keissleriella rarum* to *Lentithecium*. Subsequent phylogenetic findings [71] revealed that *L. rarum* clusters with *K. trichophoricola* within *Keissleriella*. Accordingly, *L. rarum* was reinstated in the genus *Keissleriella*. This reclassification of *Keissleriella linearis* into *Lentithecium* (as *L. lineare*) was also supported by the research of Zhang et al. [26], whose conclusions were drawn from phylogenetic analysis of LSU and SSU gene sequences. However, Singtripop et al. [72] later re-evaluated this classification, based on a re-examination of the type specimen and a novel phylogenetic analysis of the LSU locus; they reinstated the species within the genus *Keissleriella*, a taxonomic decision subsequently upheld by later phylogenetic studies [14,71,73]. Recent taxonomic revisions of *Lentithecium* species have been largely driven by evidence from multi-gene phylogenetic studies [14,71,74]. Several *Lentithecium* species, including *L. cangshanense*, *L. carbonneanum*, *L. kunmingense*, *L. unicellulare*, and *L. voraginesporum*, were transferred to *Halobyssothecium* by Calabon et al. [71]. A new monotypic genus, *Neokeissleriella*, is established within Lentitheciaceae in this study, following its isolation and identification. Notably, this genus forms a well-supported clade within Lentitheciaceae (Figure 1) with three monotypic bambusicolous genera: *Katumotoa*, *Neoophiosphaerella*, and *Pseudokeissleriella*. Nevertheless, both morphological characteristics and phylogenetic analysis support their classification as distinct genera.

Three novel species of *Keissleriella*, all obtained from grasses, were identified; although morphologically similar to other members of the genus, these taxa can be distinguished by a suite of morphological and molecular characters, which encompasses the size of ascomata, asci, and ascospores, ascospore septation patterns, host associations, and phylogenetic evidence [14,36,39,49]. Based on phylogenetic analysis, *Keissleriella* was separated into three subclades by opening them up for further studies. In our phylogenetic tree (Figure 1), species of *Keissleriella* were polyphyletic, forming two separate groups. This finding corroborates patterns previously documented in the literature, thereby strengthening the existing body of evidence [28,33,49]. It is notable that all species in Group 2 possess fusiform, pale brown, and multiseptate ascospores, whereas the majority of species in Group 1 have hyaline ascospores. A fundamental distinction in reproductive state exists between the clades: all species in Group 2 possess only the sexual morph, whereas those in Group 1 exhibit both sexual and asexual morphs. This difference thus prevents a comprehensive morphological comparison between them. Furthermore, Sun et al. [67] transferred several species—*Pleurophoma acaciae*, *P. italica*, *P. ossicola*, and *Phoma pleurospora*—into the genus *Keissleriella*, thereby enriching its species diversity. We speculate that if the future studies introduce more novel species to *Keissleriella*, consideration could be given to dividing the genus into two, based on differences in the sexual morph, asexual morph, spore color, or other characteristics.

## 5. Conclusions

A total of six grass-associated fungal species—*Neokeissleriella fusispora*, *Keissleriella caraganae*, *K. gloeospora*, *K. guttata*, *K. sichuanensis*, and *K. yunnanensis*—were identified in this study, revealing a highly diverse fungal community associated with different grasses, even when they share the same host.

## Figures and Tables

**Table 1:** The taxa used in the phylogenetic analysis and their GenBank accession numbers. Newly generated sequences are indicated with *, and the ex-type strains are in bold. “N/A” denotes the unavailable sequences.

Taxa	Vouchers/Strains	GenBank Accession Numbers			
		LSU	SSU	ITS	*tef1-α*
***Crassoascoma potentillae***	**UESTCC 21.0010**	**OK161254**	**OK161233**	**OK161237**	**OK181165**
***Crassoascoma potentillae***	**CGMCC 3.20483**	**OK161257**	**OK161236**	**OK161240**	**OK181168**
***Darksidea alpha***	**CBS 135650**	**KP184019**	**KP184049**	**KP183998**	**KP184166**
***Darksidea beta***	**CBS 135637**	**KP184023**	**KP184074**	**KP183978**	**KP184189**
***Halobyssothecium unicellulare***	**MD 6004**	**KX505376**	**KX505374**	**N/A**	**N/A**
***Halobyssothecium voraginesporum***	**MD 1342**	**KX499520**	**KX499519**	**N/A**	**N/A**
***Katumotoa bambusicola***	**KT 1517a**	**AB524595**	**AB524454**	**LC014560**	**AB539108**
***Keissleriella bambusicola***	**KUMCC 18-0122**	**MK995880**	**MK995878**	**MK995881**	**MN213156**
***Keissleriella breviasca***	**KT 649**	**AB807588**	**AB797298**	**AB811455**	**AB808567**
*Keissleriella breviasca*	KT 581	AB807587	AB797297	AB811454	AB808566
***Keissleriella camporesiana***	**MFLUCC 15-0029**	**MN401741**	**MN401743**	**MN401745**	**MN397907**
***Keissleriella camporesii***	**MFLUCC 15-0117**	**MN252886**	**MN252907**	**MN252879**	**N/A**
***Keissleriella caraganae***	**KUMCC 18-0164**	**MK359439**	**MK359444**	**MK359434**	**MK359073**
*Keissleriella caraganae* *	UESTCC 24.0218	PV394907	PV394898	PV394916	PV461187
***Keissleriella cirsii***	**MFLUCC 16-0454**	**KY497780**	**KY497782**	**KY497783**	**KY497786**
***Keissleriella cladophila***	**CBS 104.55**	**GU301822**	**GU296155**	**MH857391**	**GU349043**
*Keissleriella culmifida*	KT 2308	AB807591	AB797301	LC014561	AB808570
*Keissleriella culmifida*	KT 2642	AB807592	AB797302	LC014562	AB808571
***Keissleriella dactylidicola***	**MFLUCC 13-0866**	**KT315506**	**KT315505**	**N/A**	**KT315507**
*Keissleriella genistae*	CBS 113798	GU205222	GU205242	N/A	N/A
*Keissleriella gloeospora*	KT 829	AB807589	AB797299	LC014563	AB808568
*Keissleriella gloeospora* *	UESTCC 24.0217	PV394908	PV394899	PV394917	PV461184
***Keissleriella guttata*** *	**CGMCC 3.28492**	**PV394904**	**PV394895**	**PV394913**	**PV461183**
*Keissleriella guttata* *	UESTCC 24.0214	PV394905	PV394896	PV394914	PV461184
*Keissleriella linearis*	MFLUCC 19-0410	MN598873	MN598870	MN598892	MN607978
*Keissleriella linearis*	MFLUCC 20-0224	MT068487	MT068492	MT232436	MT477866
*Keissleriella phragmiticola*	CPC 33249	MT223903	N/A	MT223808	MT223715
***Keissleriella phragmiticola***	**MFLUCC 17-0779**	**MG829014**	**N/A**	**MG828904**	**N/A**
*Keissleriella poagena*	CBS 136767	KJ869170	N/A	KJ869112	N/A
*Keissleriella rara*	CBS 118429	GU479791	GU479757	N/A	N/A
***Keissleriella rosacearum***	**MFLUCC 15-0045**	**MG829015**	**MG829123**	**N/A**	**N/A**
***Keissleriella rosae***	**MFLUCC 15-0180**	**MG829016**	**MG922549**	**N/A**	**N/A**
***Keissleriella rosarum***	**MFLUCC 15-0089**	**MG829017**	**MG829124**	**MG828905**	**N/A**
***Keissleriella sichuanensis*** *	**CGMCC 3.28490**	**PV394902**	**PV394893**	**PV394911**	**PV461180**
*Keissleriella sichuanensis* *	UESTCC 24.0212	PV394903	PV394894	PV394912	PV461182
***Keissleriella sparticola***	**MFLUCC 14-0196**	**KP639571**	**N/A**	**N/A**	**N/A**
***Keissleriella sporoboli***	**BRIP:63688**	**NG_088212**	**N/A**	**MW682816**	**OM001125**
***Keissleriella tamaricicola***	**MFLUCC 14-0168**	**KU900300**	**N/A**	**KU900328**	**N/A**
***Keissleriella quadriseptata***	**KT 2292**	**AB807593**	**AB797303**	**AB811456**	**AB808572**
***Keissleriella taminensis***	**KT 571**	**AB807595**	**AB797305**	**LC014564**	**AB808574**
***Keissleriella trichophoricola***	**CBS 136770**	**KJ869171**	**N/A**	**KJ869113**	**N/A**
***Keissleriella yonaguniensis***	**KT 2604**	**AB807594**	**AB797304**	**AB811457**	**AB808573**
***Keissleriella yunnanensis***	**HKAS 136902**	**PQ569964**	**PQ651970**	**PQ324187**	**PQ761123**
*Keissleriella yunnanensis*	UESTCC 24.0216	PV394906	PV394897	PV394915	PV461184
*Keissleriella sp.*	KT895	AB807590	AB797300	N/A	AB808569
***Lentithecium clioninum***	**KT 1149A**	**AB807540**	**AB797250**	**LC014566**	**AB808515**
***Lentithecium pseudoclioninum***	**KT 1113**	**AB807545**	**AB797255**	**AB809633**	**AB808521**
***Murilentithecium clematidis***	**MFLUCC 14-0562**	**KM408759**	**KM408761**	**KM408757**	**KM454445**
***Murilentithecium lonicerae***	**MFLUCC 18-0675**	**MK214373**	**MK214376**	**MK214370**	**MK214379**
***Neokeissleriella fusispora*** *	**CGM** **CC** **3** **.** **28674**	**PV394900**	**PV394891**	**PV394909**	**PV461179**
*Neokeissleriella fusispora* *	UESTCC 24.0210	PV39490	PV394892	PV394910	PV461180
***Neoophiosphaerella sasicola***	**KT 1706**	**AB524599**	**AB524458**	**LC014577**	**AB539111**
***Paralentithecium suae***	**CGMCC 3.24265**	**OQ732683**	**OQ875040**	**OQ874972**	**OR367672**
***Paralentithecium aquaticum***	**CBS 123099**	**GU301823**	**GU296156**	**NR_160229**	**GU349068**
***Phragmocamarosporium platani***	**MFLUCC 14-1191**	**KP842916**	**KP842919**	**N/A**	**N/A**
***Phragmocamarosporium rosae***	**MFLUCC 17-0797**	**MG829051**	**MG829156**	**N/A**	**MG829225**
***Pleurophoma ossicola***	**CPC 24985**	**KR476770**	**N/A**	**KR476737**	**N/A**
***Pleurophoma ossicola***	**CBS 139905**	**KR476769**	**N/A**	**KR476736**	**N/A**
***Pleurophoma italica***	**MFLUCC 15-0061**	**N/A**	**KY501122**	**N/A**	**KY514398**
***Pleurophoma acaciae***	**CPC 29188**	**KY173524**	**N/A**	**KY173434**	**N/A**
***Pleurophoma pleurospora***	**CBS 130329**	**JF740327**	**N/A**	**N/A**	**N/A**
***Poaceascoma halophila***	**MFLUCC 15-0949**	**MF615399**	**MF615400**	**N/A**	**N/A**
***Poaceascoma taiwanense***	**MFLU 18-0083**	**MG831567**	**MG831568**	**MG831569**	**N/A**
***Pseudokeissleriella bambusicola***	**CGMCC 3.20950**	**ON614138**	**ON614096**	**ON614135**	**ON639623**
*Pseudokeissleriella bambusicola*	UESTCC 22.0028	ON614137	ON614095	ON614134	ON639622
*Setoseptoria magniarundinacea*	KT 1174	AB807576	AB797286	LC014596	AB808552
*Stagonospora macropycnidia*	CBS 114202	GU301873	GU296198	N/A	GU349026
***Setoseptoria phragmitis***	**CBS 114802**	**KF251752**	**N/A**	**KF251249**	**N/A**
***Tingoldiago clavata***	**MFLUCC 19-0496**	**MN857178**	**MN857186**	**MN857182**	**N/A**
***Tingoldiago hydei***	**MFLUCC 19-0499**	**MN857177**	**N/A**	**MN857181**	**N/A**
***Towyspora aestuari***	**MFLUCC 15-1274**	**KU248852**	**KU248853**	**KU248851**	**N/A**
*Wettsteinina lacustris*	CBS 618.86	N/A	DQ678023	AF250831	DQ677919
*Massarina cisti*	CBS 266.62	FJ795447	FJ795490	LC014568	AB808514
*Massarina eburnea*	CBS 473.64	GU301840	GU296170	AF383959	GU349040

## Data Availability

The data presented in this study are openly available in National Center for Biotechnology Information at A1098531023601210.
